# Breast cancer clinical trial participation among diverse patients at a comprehensive cancer center

**DOI:** 10.1038/s41523-024-00672-0

**Published:** 2024-08-03

**Authors:** Emily L. Podany, Shaun Bulsara, Katherine Sanchez, Kristen Otte, Matthew J. Ellis, Maryam Kinik

**Affiliations:** 1 Baylor College of Medicine, Lester and Sue Smith Breast Center, Houston, TX USA; 2 Washington University in St. Louis, St. Louis, MO USA; 3 The Institute for Proteogenomic Discovery, Houston, TX USA

**Keywords:** Breast cancer, Public health

## Abstract

This study was designed to determine the enrollment patterns in breast cancer clinical trials (CCTs) of patients with diverse backgrounds in an equal access setting and to evaluate the factors contributing to low rates of clinical trial accrual in patients of low socioeconomic status (SES). We performed a retrospective review of a prospectively maintained database of new patients seen at the Dan L. Duncan Comprehensive Cancer Center dating from 5/2015 to 9/2021, which included 3043 patients screened for breast CCTs. We compared the rate of CCT availability, eligibility, and enrollment between two patient populations: Smith Clinic, where most patients are of low SES and uninsured, and Baylor St. Luke’s Medical Center (BSLMC) with mostly predominantly insured, higher income patients. We performed logistic regression to evaluate whether differences in age, clinic, race, trial type, and primary language may be underlying the differences in CCT enrollment. More patients were eligible for CCTs at Smith Clinic (53.7% vs 44.7%, *p* < 0.001). However, Smith Clinic patients were more likely to decline CCT enrollment compared to BSLMC (61.3% declined vs 39.4%, *p* < 0.001). On multivariate analysis, Black patients had a significantly higher rate of CCT refusal overall (OR = 0.26, 95% CI 0.12–0.56, *p* < 0.001) and BSLMC only (OR = 0.20, 95% CI 0.060–0.60, *p* = 0.006). Our data shows that it is likely an oversimplification to assume that equal access will lead to the elimination of CCT disparities. Efforts to diversify CCTs must include consideration of structural and institutional inequities as well as social needs.

## Introduction

Black cancer patients in the United States have both increased overall cancer mortality and increased cancer-specific mortality^1–3^. In breast cancer, Black women have a 41% higher risk of dying from breast cancer when compared with White women and present on average at a later stage^2,4,5^. Structural inequities pertaining to access to care, diagnosis timing, and treatment delay affect Black women disproportionately^6,7^. While these are socioeconomic predictors of the observed poor outcomes, it is also well documented that Black women have a higher incidence of more aggressive breast cancer subtypes (i.e., triple negative breast cancer (TNBC)) than any other ethnic or racial groups^8^. It is critical to understand the biological basis of the observed poor outcomes of breast cancer among Black women. As we work to design precision-driven interventions for prevention, timely diagnosis, and treatment, achieving cancer health equity is not feasible without improving diversity in cancer clinical trials (CCTs).

In an ideal world, the populations studied in CCTs would be representative of the diversity of patients seen in clinic, and CCTs would be used as a tool to decrease inequity. Unfortunately, well-documented disparities exist within CCTs. The National Institutes of Health (NIH) Revitalization Act of 1993 aimed to increase the number of women and underrepresented racial groups in clinical research through mandated inclusion, yet numbers remain low^9^. In 2014, only approximately 1% of NCI-sponsored clinical trials were primarily focused on racial and ethnic minorities^10^. Studies have shown that the average enrollment of Black Americans in CCTs is at best between 5 and 7%, despite Black Americans making up more than 13% of the general population of the United States^11–13^.

When access to CCTs is not a barrier to enrollment, the rate of clinical trial participation by racial and ethnic minorities, especially those of low SES, has not been well studied. Data often comes from safety-net hospitals or private institutions, but rarely are both serving the same catchment area. The Dan L. Duncan Comprehensive Cancer Center (DLDCCC) in Houston, Texas provides access to breast CCTs at two clinical sites: Smith Clinic (SC), within the safety-net Harris Health System, and Baylor St. Luke’s Medical Center (BSLMC). We hypothesized that the racial and socioeconomic gap in clinical trial enrollment would be at least partially improved by similar access to breast CCTs at the two sites.

## Results

### Eligibility for CCTs

**Fig. 1:**
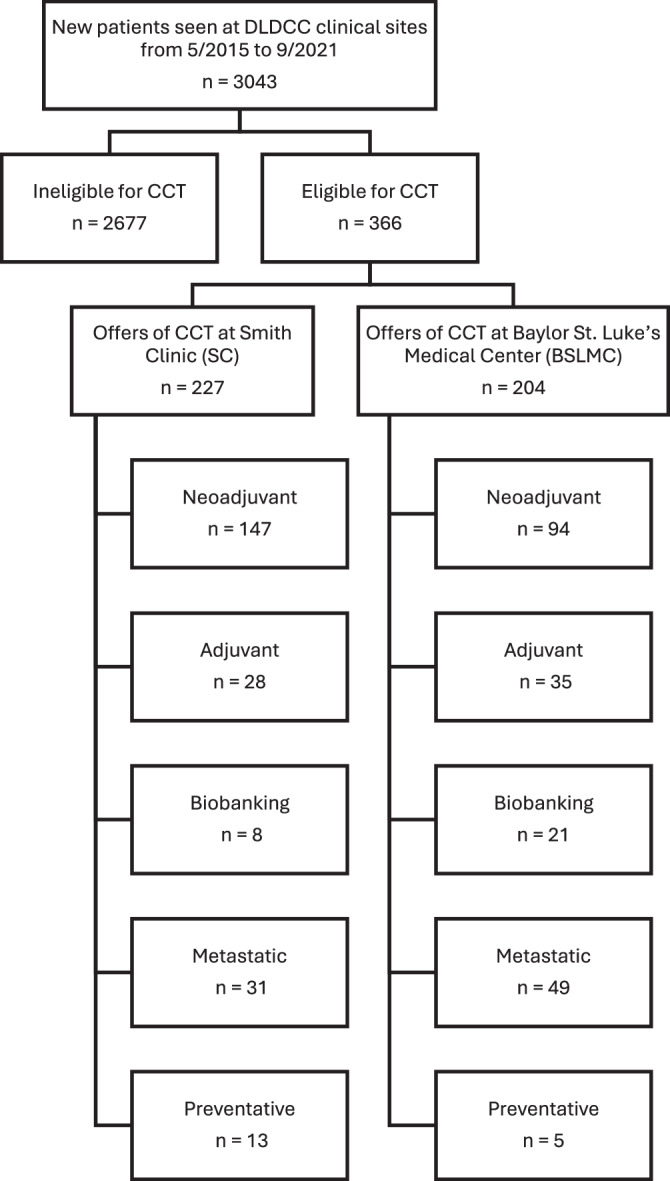
Consort diagram for the retrospective cohort study. We identified 3043 new patients seen at DLDCC clinical sites between 5/2015 and 9/2021, 366 of whom were eligible for CCT. The majority of patients at each site were eligible for neoadjuvant trials and patients were often eligible for more than one CCT.

Of the 3043 patients screened for breast CCTs, 366 patients were found to be eligible for CCT, and some patients were eligible for multiple CCTs. There were 431 total offers to CCTs (Fig. 1).

The patient demographics of the 3043 new patients seen at the DLDCCC and screened for breast CCT eligibility from 5/2015 to 7/2021 are shown in Table 1. Notably, 50% of these patients were White at BSLMC in comparison to 11% at SC, and 74% listed English as their primary language at BSLMC versus 47% at SC. Patients at SC were on average younger, and more frequently presenting with TNBC compared to BSLMC.

**Table 1:** Demographics of the patient populations at SC and BSLMC including race, primary spoken language, and ethnicity

	Screened (*n* = 3043)		Eligible (*n* = 366)	
	BSLMC (*n* = 1643)	SC (*n* = 1400)	BSLMC (*n* = 175)	SC (*n* = 191)
Age in years [Mean (SD)]	56.3 (12.8)	54.5 (11.0)	55.8 (11.6)	53.7 (10.2)
Race				
White/Caucasian	837 (50.9%)	165 (11.8%)	99 (56.6%)	26 (13.6%)
Black/African-American	157 (9.6%)	392 (28.0%)	18 (10.3%)	42 (22.0%)
Hispanic/Latino	35 (2.1%)	544 (38.9%)	2 (1.1%)	92 (48.2%)
Asian/Pacific Islander	21 (1.3%)	57 (4.1%)	2 (1.1%)	4 (2.1%)
American Indian	10 (0.6%)	2 (0.1%)	1 (0.6%)	0 (0.0%)
Other	167 (10.2%)	194 (13.9%)	16 (9.1%)	22 (11.5%)
Unknown	416 (25.3%)	46 (3.3%)	37 (21.1%)	5 (2.6%)
Primary Language				
English	1217 (74.1%)	667 (47.6%)	135 (77.1%)	76 (39.8%)
Spanish	41 (2.5%)	601 (42.9%)	4 (2.3%)	110 (57.6%)
Vietnamese	8 (0.5%)	45 (3.2%)	1 (0.6%)	1 (0.5%)
Other	17 (1.0%)	76 (5.4%)	2 (1.1%)	3 (1.6%)
Unknown	360 (21.9%)	11 (0.8%)	33 (18.9%)	1 (0.5%)
Ethnicity				
Hispanic/Latino	523 (31.8%)	739 (52.8%)	48 (27.4%)	120 (62.8%)
Non-Hispanic/Latino	1057 (64.3%)	636 (45.4%)	122 (69.7%)	69 (36.1%)
Unknown	63 (3.8%)	25 (1.8%)	5 (2.9%)	2 (1.0%)
Subtype				
ER+	905 (55.1%)	768 (54.9%)	114 (65.1%)	99 (51.8%)
PR+	761 (46.3%)	655 (46.8%)	91 (52.0%)	89 (46.6%)
HER2+	336 (20.5%)	239 (17.1%)	51 (29.1%)	51 (26.7%)
TNBC	132 (8.0%)	190 (13.6%)	25 (14.3%)	56 (29.3%)
Available for Trial Enrollment	734 (44.7%)	752 (53.7%)	N/A	N/A
Eligible for Trial Enrollment	175 (10.7%)	191 (13.6%)	N/A	N/A
Accepted Trial Enrollment (among Eligible)	N/A	N/A	106 (60.6%)	74 (38.7%)

More patients at SC had a trial available to them (752/1400, 53.7%) versus at BSLMC (734/1643, 44.7%, *p*-value < 0.001) (Table 1). Patients at SC were also more likely to be eligible for CCTs (191/1400, 13.6%) than patients at BSLMC (175/1643, 10.7%, *p*-value = 0.011).

### Enrollment patterns in CCTs

Despite higher eligibility, patients at SC were less likely to accept these CCT offers (74/191 accepted, 38.7%) than patients at BSLMC (106/175 accepted, 60.6%, p-value < 0.001) (Table 1). This difference in acceptance was significant on univariate but not multivariate analysis (Table 2). Age was not found to be significantly associated with trial enrollment.

**Table 2:** Results of multivariable analysis by age, clinic site, race, trial category, and spoken language

Demographic	Declined *N* = 241	Accepted *N* = 190	Unadjusted OR, 95CI (*p*-value)	Adjusted* OR, 95CI (*p*-value)
Age				
20–30 years	2 (0.8%)	3 (1.6%)	2.100 (0.320, 17.075) *p* = 0.439	0.793 (0.050, 22.606) *p* = 0.874
31–40 years (Ref)	28 (11.6%)	20 (10.5%)	N/A	N/A
41–50 years	55 (22.8%)	37 (19.5%)	0.942 (0.464, 1.927) *p* = 0.868	1.044 (0.461, 2.395) *p* = 0.918
51–60 years	62 (25.7%)	56 (29.5%)	1.104 (0.566, 2.182) *p* = 0.773	1.201 (0.550, 2.674) *p* = 0.648
61–70 years	71 (29.5%)	53 (27.9%)	1.197 (0.608, 2.385) *p* = 0.605	0.950 (0.419, 2.174) *p* = 0.902
71–80 years	8 (3.3%)	12 (6.3%)	2.100 (0.734, 6.276) *p* = 0.171	2.008 (0.568, 7.590) *p* = 0.287
81+ years	2 (0.8%)	1 (0.5%)	0.700 (0.031, 7.798) *p* = 0.777	0.354 (0.011, 6.208) *p* = 0.488
Clinic				
BSLMC (Ref)	88 (36.5%)	116 (61.1%)	N/A	N/A
SC	153 (63.5%)	74 (38.9%)	**0.367 (0.247, 0.542)** ***p*** < **0.001**	0.602 (0.321, 1.125) *p* = 0.111
Race				
White/Caucasian (Ref)	53 (22.0%)	72 (37.9%)	N/A	N/A
Asian/Pacific Islander	10 (4.1%)	10 (5.3%)	0.736 (0.283, 1.916) *p* = 0.525	0.843 (0.293, 2.442) *p* = 0.750
Black/African-American	49 (20.3%)	18 (9.5%)	**0.270 (0.139, 0.509)** ***p*** < **0.001**	**0.261 (0.116, 0.563)** ***p*** < **0.001**
Hispanic/Latino	104 (43.2%)	60 (31.6%)	**0.425 (0.263, 0.682)** ***p*** < **0.001**	0.763 (0.319, 1.815) *p* = 0.539
Other	2 (0.8%)	11 (5.8%)	4.049 (1.032, 26.871) *p* = 0.077	**7.361 (1.338, 72.617)** ***p*** = **0.042**
Trial Category				
Neoadjuvant (Ref)	150 (62.2%)	91 (47.9%)	N/A	N/A
Adjuvant	38 (15.8%)	25 (13.2%)	1.084 (0.609, 1.905) *p* = 0.780	0.897 (0.451, 1.756) *p* = 0.753
Biobanking	4 (1.7%)	25 (13.2%)	**10.302 (3.849, 35.825)** ***p*** < **0.001**	**12.799 (3.777, 61.403)** ***p*** < **0.001**
Metastatic	40 (16.6%)	40 (21.1%)	1.648 (0.990, 2.750) *p* = 0.055	1.542 (0.833, 2.858) *p* = 0.167
Preventative	9 (3.7%)	9 (4.7%)	1.648 (0.622, 4.370) *p* = 0.308	**4.785 (1.214, 23.858)** ***p*** = **0.033**
Language				
English (Ref)	127 (52.7%)	123 (64.7%)	N/A	N/A
Spanish	89 (36.9%)	46 (24.2%)	**0.534 (0.344, 0.820)** ***p*** = **0.046**	0.759 (0.322, 1.832) *p* = 0.532
Other	7 (2.9%)	2 (1.1%)	0.295 (0.043, 1.249) *p* = 0.133	0.184 (0.019, 1.069) *p* = 0.087

^*^ORs adjusted for all other variables in the table. **Bold indicates statistical significance (*p*** < **=0.05)**.

Univariate analysis of the patients showed that Black patients, Hispanic/Latino patients, and Spanish speaking patients were significantly more likely to decline CCT participation. However, on overall multivariate analysis, only the Black patient category was associated with significantly higher rate of enrollment refusal (odds ratio (OR) = 0.26, 95% CI 0.12–0.56, *p* < 0.001). (Table 2) On the multivariate analyses across the two clinical sites, patients were significantly more likely to accept biobanking trials than other trial types at SC (OR = 16.90, 95% CI 2.13–363.77, *p* = 0.018) and at BSLMC (OR = 20.10, 95% CI 3.37–395.53, *p*-value = 0.007). Patients at SC were also more likely to enroll into preventive trials (OR = 7.88, 95% CI 1.53–59.39, p-value = 0.020). Primary language was not found to be a determining factor in trial enrollment or refusal at either site. Black patients at BSLMC were less likely to enroll in CCTs (OR = 0.20, 95% CI 0.060–0.60, *p* = 0.006) on multivariate analysis, however this was not significant on multivariate analysis in the SC subset (OR = 0.41, 95% CI 0.11–1.53, p = 0.180). (Supplemental Tables 1 and 2).

## Discussion

While patients at SC have equal opportunity when it comes to access to clinical trials, trial enrollment is only at 37% in this clinic site, compared to BSLMC clinic where over 61% of trial eligible patients consent to enrollment. Overall, Black patients were less likely to consent to trial enrollment, and the rate of trial refusal was lowest for biobanking trials across both sites and preventive trials at SC compared to other trials. Speaking a primary language other than English was not found to be a major barrier to enrollment in our population.

Our data shows that it is likely an oversimplification to assume that equal access will lead to a complete elimination of CCT disparities. At SC, which serves a more diverse population with a higher percentage of low income and uninsured patients, the patients were significantly more eligible for breast CCTs. As noted in Table 1, patients at SC had a higher rate of TNBC (29.3% versus 14.3% at BSLMC), which we hypothesize may be one reason for the higher rate of eligibility. More SC patients were eligible for neoadjuvant trials and biobanking trials than at BSLMC (Fig. 1), possibly also due to the higher TNBC rates in this population.

Despite the higher rate of eligibility at SC, these patients were significantly more likely to decline the CCT. Our findings in a highly racially and ethnically diverse patient population supports the literature that shows that Black patients are less likely to agree to participate in clinical trials. The causes of discrepancy between eligibility and enrollment are multifactorial and complex. Studies have shown equal willingness among patients of different races to participate in clinical trials^14–17^, yet disparities in enrollment persist. In 2008, Ford^18^ identified three categories of reasons for low accrual: awareness, opportunity, and acceptance/refusal barriers or promoters.

Awareness barriers include lack of knowledge about the purpose and availability of CCTs^13,19^. Cancer health literacy has been found in some studies to be significantly lower in Black patients^14,20,21^, though others found that the role of factual knowledge did not make a significant difference in accrual^22^. The FDA in 2020 published guidelines and potential approaches to increase the diversity of clinical trial populations, including making diversity of enrollment a priority, involving the community, and educating potential participants^23^. When CCTs do not recruit a diverse patient population and fail to be made available to racial or ethnic minorities^17^, the results cannot be assumed to be generalizable to the community at large.

Opportunity barriers include limitations due to socioeconomic status and ineligibility. Research has shown us that CCT participants are less likely to be Black and more likely to be of a higher socioeconomic status^24–28^. Black patients are more likely to be deemed ineligible for clinical trials^13,16,29^. This is partially due to a higher rate of comorbidities such as hypertension, vision loss, or diabetes, as well as benign neutropenia—a condition that has not been shown to increase risk of infection^16^. However, studies have also shown that Black Americans are more likely to be deemed ineligible due to perceived noncompliance or mental status, and that subjective judgements on eligibility more often favor White patients^29^.

Barriers to acceptance include an understandable mistrust in a medical system that has historically caused harm to people of color, perceived financial burden, logistical difficulties including transportation, and family or cultural pressures. When Black American patients are asked about their reasons for opting out of CCT, studies show us that a lack of trust is one of the most common factors influencing their decision^13,22^. Barriers relating to logistics or finances are seen more often in safety-net hospitals and clinics^19^.

At BSLMC, where the population is less diverse, we noted a difference in CCT enrollment by race in multivariate analysis. However, this finding was not significant at SC, which has a more diverse population (Supplemental Tables 1 and 2). This is an interesting exploratory finding that can be further elucidated in future studies but may point to more diverse clinic experiences encouraging CCT enrollment. This could be due to higher trust in the clinic, awareness of clinical trials, or physicians offering trials more equitably. A limitation of our finding is the low number of patients in each category, and future studies would need to clarify these findings with a larger patient population.

Although it is imperative that we continue to shine a light on these important issues, we must be ready to envision and enact both local and national policy changes. Moving forward, we are focusing on community engagement, patient education, and dialogue with our patients to explore specific interventions designed to improve our Black patient population’s views of trial enrollment. Interventions have been attempted around the country to varying levels of success, including patient navigation systems^30–32^, patient education videos^12,13^, the recent ACCURE trial which included multiple levels of intervention including electronic medical record changes and specific physician roles^33^, diversifying staff, ensuring trial resources are in multiple languages, and offering financial incentives^23^. The reason for trial refusal was unfortunately not uniformly captured in the clinical trial database nor in patients’ electronic medical records. This is a limitation of our study; we do not have specific patients’ refusal reasons. In a follow up study that is currently being conducted, we have designed a patient education intervention to collect specific information on patients’ attitudes towards clinical trial enrollment and refusal. This follow-up study will serve as a roadmap for designing patient and community targeted outreach programs to improve our trial enrollment.

Cancer clinical trials have maintained restrictive eligibility criteria that inevitably censor out a large population of patients^34^. It is crucial that efforts continue on all fronts to improve cancer clinical trial diversity, including clinical trial design and challenging long-standing beliefs on eligibility criteria. Prevention and treatment alike need to be considered when designing an equitable future for cancer care, and as others have shown, these efforts must include consideration of structural and institutional inequities as well as social needs. Research and data collection are only the first steps in a necessary journey toward equity in cancer care.

## Methods

### Study population and data collection

This is a retrospective cohort study of new patients seen from 5/2015 to 9/2021, which included 3043 patients screened for breast CCTs at DLDCCC clinical sites. The populations receiving care at the two clinical sites differ greatly. At SC, half of the patients earn less than $25,000 annually, 60% are uninsured and use a county financial assistance program known as the “Gold Card,” and 65% are not proficient in English. Fifty-nine percent of SC patients self-identify as Hispanic and 29% self-identify as Black, with White patients making up 10% of the population. At BSLMC, over 95% of the patients have federal and commercial insurance and 68% are White, 13% are Black, and 3% are Hispanic. We collected information on age at the time of screening for CCTs, patient-reported race, and primary spoken language.

The study was conducted according to the ethical guidelines set forth in the Declaration of Helsinki and in concordance with the Heath Insurance Portability and Accountability Act. The study was approved by the institutional review board (IRB) of Baylor College of Medicine. The requirement of patient informed consent was waived by the IRB as the data was deidentified prior to analysis.

DLDCCC is an active participant in several cooperative group consortia including the Translational Breast Cancer Research Consortium (TBCRC), Southwest Oncology Group (SWOG), and NRG oncology (from the parental organizations of NSABP, RTOG, and GOG), and it is frequently the leading site for national investigator initiated clinical trials (IIT). We collected information on whether there were trials available for the patients’ diagnosis, trial eligibility, and the type of trial the patients were screened for. We designated 5 categories of trials based on the intent of the trial (e.g., scalp cooling trial to prevent chemotherapy-associated hair loss) and the stage of therapy that the trial was offered (e.g., COMPASS RD, an adjuvant trial). The categories were preventive, neoadjuvant, adjuvant, metastatic, and biobanking. The same CCTs were open at both sites under the DLDCCC.

### Statistical analysis

We first performed Chi-square tests to determine whether there were differences in trial availability, trial eligibility, and trial acceptance rate according to DLDCCC clinical sites. We then performed univariate and multivariate logistic regression to evaluate whether differences in age, clinic site, race, trial type, and primary language may be underlying the observed differences in CCT enrollment rates. We performed logistic regression on the overall dataset as well as by clinic. We calculated odds ratios with 95% confidence intervals to measure the strength of association between the predictors and enrollment. P-values less than 0.05 were considered statistically significant. Analysis was performed using R version 4.1.0.

### Supplementary information

Supplemental tables 1 and 2

## Data Availability

The participants in this study did not give written consent for their data to be shared. Due to the clinical nature of the dataset, it is not available publicly.
