# Supplementary material for: Breast cancer clinical trial participation among diverse patients at a comprehensive cancer center
Source: NPJ Breast Cancer. 2024 Aug 3;10:70. doi: 10.1038/s41523-024-00672-0 (PMC11297908; doi:10.1038/s41523-024-00672-0)
Supplement: Supplementary file 1 — Supplemental tables 1 and 2 [file 41523_2024_672_MOESM1_ESM.pdf]

Supplemental table 1: Results of multivariable analysis of SC patients by age, race, trial category, and spoken language

Demographic	Declined<br>N = 153	Accepted<br>N = 74	Unadjusted OR, 95CI (p-value)	Adjusted* OR, 95CI (p-value)
Age				
20-30 years	1 (0.7%)	0 (0.0%)	N/A[1]	N/A[1]
31-40 years (Ref)	21 (13.7%)	7 (9.5%)	N/A	N/A
41-50 years	34 (22.2%)	19 (25.7%)	1.676 (0.620, 4.907) p=0.322	1.732 (0.586, 5.616) p=0.335
51-60 years	44 (28.8%)	25 (33.8%)	1.705 (0.657, 4.834) p=0.289	2.262 (0.775, 7.302) p=0.149
61-70 years	35 (22.9%)	17 (23.0%)	1.457 (0.532, 4.298) p=0.475	1.415 (0.435, 4.896) p=0.570
71-80 years	3 (2.0%)	2 (2.7%)	2.000 (0.228, 14.687) p=0.493	1.944 (1.885, 16.885) p=0.547
81+ years	2 (1.3%)	0 (0.0%)	N/A[1]	N/A[1]
Race				
White/Caucasian (Ref)	17 (11.1%)	7 (9.5%)	N/A	N/A
Asian/Pacific Islander	2 (1.3%)	2 (2.7%)	2.429 (0.251, 23.817) p=0.418	2.876 (0.187, 78.788) p=0.458
Black/African-American	34 (22.2%)	11 (14.9%)	0.786 (0.260, 2.470) p=0.671	0.415 (0.112, 1.530) p=0.182
Hispanic/Latino	96 (62.7%)	50 (67.6%)	1.265 (0.509, 3.455) p=0.626	1.269 (0.306, 5.326) p=0.741
Other	1 (0.7%)	4 (5.4%)	9.714 (1.182, 209.293) p=0.059	N/A[1]
Trial Category				
Neoadjuvant (Ref)	106 (69.3%)	41 (55.4%)	N/A	N/A
Adjuvant	21 (13.7%)	7 (9.5%)	0.862 (0.319, 2.097) p=0.754	0.809 (0.258, 2.276) p=0.698
Biobanking	3 (2.0%)	5 (6.8%)	4.309 (1.012, 21.782) p=0.052	**16.902 (2.134, 363.768) p=0.018**
Metastatic	16 (10.5%)	15 (20.3%)	**2.424 (1.092, 5.375) p=0.028**	2.204 (0.896, 5.407) p=0.082
Preventative	7 (4.6%)	6 (8.1%)	2.216 (0.677, 7.060) p=0.175	**7.876 (1.534, 59.390) p=0.020**
Language				
English (Ref)	60 (39.7%)	30 (40.5%)	N/A	N/A
Spanish	87 (56.9%)	43 (58.1%)	0.989 (0.560, 1.757) p=0.968	0.646 (0.219, 2.008) p=0.434
Other	4 (2.6%)	1 (1.4%)	0.500 (0.025, 3.565) p=0.543	N/A[1]

ORs adjusted for all other variables in the table. [1]Group sizes were not large enough to perform comparison.* **Bold indicates statistical significance (p<=0.05).

Supplemental table 2: Results of multivariable analysis of BSLMC patients by age, race, trial category, and spoken language

Demographic	Declined N = 88	Accepted N = 116	Unadjusted OR, 95CI (p-value)	Adjusted* OR, 95CI (p-value)
Age				
20-30 years	1 (1.1%)	3 (2.6%)	1.615 (0.168, 36.120) p=0.700	N/A[1]
31-40 years (Ref)	7 (8.0%)	13 (11.2%)	N/A	N/A
41-50 years	21 (23.9%)	18 (15.5%)	0.462 (0.145, 1.378) p=0.174	0.510 (0.114, 2.084) p=0.358
51-60 years	27 (30.7%)	31 (26.7%)	0.618 (0.206, 1.738) p=0.371	0.466 (0.116, 1.684) p=0.255
61-70 years	27 (30.7%)	36 (31.0%)	0.718 (0.241, 2.002) p=0.535	0.500 (0.123, 1.828) p=0.308
71-80 years	5 (5.7%)	10 (8.6%)	1.078 (0.262, 4.611) p=0.918	1.191 (0.194, 8.222) p=0.852
81+ years	0 (0.0%)	1 (0.9%)	N/A[1]	N/A[1]
Race				
White/Caucasian (Ref)	36 (40.9%)	65 (56.0%)	N/A	N/A
Asian/Pacific Islander	8 (9.1%)	8 (6.7%)	0.554 (0.188, 1.624) p=0.275	0.645 (0.191, 2.145) p=0.469
Black/African-American	15 (17.0%)	7 (6.0%)	**0.258 (0.091, 0.672) p=0.007**	**0.203 (0.059, 0.598) p=0.006**
Hispanic/Latino	8 (9.1%)	10 (8.6%)	0.692 (0.251, 1.961) p=0.478	0.659 (0.191, 2.247) p=0.500
Other	1 (1.1%)	7 (6.0%)	3.877 (0.653, 73.966) p=0.213	1.957 (0.220, 43.175) p=0.584
Trial Category				
Neoadjuvant (Ref)	44 (50.0%)	50 (43.1%)	N/A	N/A
Adjuvant	17 (19.3%)	18 (15.5%)	0.932 (0.427, 2.037) p=0.859	1.067 (0.413, 2.762) p=0.892
Biobanking	1 (1.1%)	20 (17.2%)	**17.600 (3.447, 322.262) p=0.006**	**20.088 (3.370, 395.532) p=0.007**
Metastatic	24 (27.3%)	25 (21.6%)	0.917 (0.458, 1.834) p=0.805	1.183 (0.481, 2.946) p=0.715
Preventative	2 (2.3%)	3 (2.6%)	1.320 (0.210, 10.367) p=0.766	2.108 (0.176, 51.144) p=0.569
Language				
English (Ref)	67 (76.1%)	93 (80.2%)	N/A	N/A
Spanish	2 (2.3%)	3 (2.6%)	1.081 (0.175, 8.373) p=0.933	1.400 (0.163, 14.197) p=0.759
Other	3 (3.4%)	1 (0.9%)	0.240 (0.012, 1.921) p=0.221	0.342 (0.016, 3.094) p=0.378

ORs adjusted for all other variables in the table. [1]Group sizes were not large enough to perform comparison.* **Bold indicates statistical significance (p<=0.05).
